# Resolution of Osteoporosis in a Breast Cancer Survivor Following Supervised Osteogenic Loading and High-Adherence Lifestyle Modification: A Case Report

**DOI:** 10.7759/cureus.115096

**Published:** 2026-08-24

**Authors:** Youssef Salama, Nahla Heikal

**Affiliations:** 1 School of Medicine, Wayne State University, Detroit, USA; 2 Pathology and Laboratory Medicine, Wayne State University School of Medicine, Detroit, USA

**Keywords:** bone mineral density, breast cancer survivor, lifestyle medicine, osteogenic loading, osteoporosis, postmenopausal

## Abstract

Breast cancer survivors are at elevated risk for osteoporosis due to chemotherapy-induced ovarian dysfunction, prolonged endocrine therapy, and premature menopause, and anti-resorptive therapy remains the standard of care even though some patients decline pharmacologic treatment. We describe a 55-year-old postmenopausal woman, treated 19 years earlier for stage II hormone receptor-positive, human epidermal growth factor receptor 2-positive ductal carcinoma of the right breast, who developed osteoporosis on routine screening. Treatment had included neoadjuvant chemotherapy, modified radical mastectomy, regional nodal irradiation, one year of trastuzumab, and 10 years of tamoxifen. A scan at age 41 had shown bone density within the expected range for age, but a repeat scan on Day -20 (relative to the start of the lifestyle intervention, defined as Day 0) demonstrated osteoporosis, with a total lumbar spine *T*-score of -2.5, femoral neck *T*-score of -2.1, and total hip *T*-score of -1.9. She declined alendronate and began a structured lifestyle program on Day 0 consisting of weekly supervised osteogenic loading, progressive resistance training, increased aerobic activity, balance and mobility work, a whole-food plant-forward diet, and supplementation. A follow-up scan on Day 486 demonstrated a 4.3% increase in lumbar spine bone mineral density (*T*-score -2.2), a 4.7% increase at the femoral neck (*T*-score -1.8), and a 2.0% increase at the total hip (*T*-score -1.8), with the most severely affected vertebrae recovering the most; calcium and vitamin D remained within target ranges throughout. Over approximately 16 months without anti-resorptive medication, this patient’s lumbar spine moved from the osteoporotic to the osteopenic range, with concordant improvement at the femoral neck. Causality cannot be attributed to any single component of the intervention, but the case supports further study of structured lifestyle-based bone health strategies in breast cancer survivorship.

## Introduction

Osteoporosis remains a major contributor to morbidity and healthcare costs worldwide. About half of women and one in five men older than 50 years in the United States will sustain a fragility fracture, with a meaningful impact on survival and care costs [1,2]. Bone loss is most rapid around the final menstrual period: in naturally menopausal women, vertebral losses from one year before to two years after the final period have been reported at approximately 7.4% at the lumbar spine and 5.8% at the femoral neck [3,4]. United States Preventive Services Task Force guidance supports screening women aged 65 years and older and younger postmenopausal women at elevated risk, although the burden of low bone density in women aged 50 to 64 years is less well characterized [5,6].

Breast cancer survivors carry a particularly high risk [7]. Cytotoxic chemotherapy can impair osteoblast function and accelerate resorption, and chemotherapy-induced ovarian failure may produce an abrupt drop in estrogen with rapid declines in bone mass [7,8]. The effect of tamoxifen on bone is complex and depends on menopausal status: in premenopausal patients, it can act as a partial estrogen antagonist with adverse skeletal effects, while in postmenopausal patients, it is generally bone-protective [9-11]. The American Society of Clinical Oncology therefore recommends periodic bone density assessment in survivors at substantial risk for treatment-related bone loss [12].

Estrogen is central to skeletal homeostasis. It suppresses osteoclastogenesis, supports osteoblast activity, and balances resorption with formation. With menopause, declining estrogen accelerates resorption and shifts the immune milieu toward a more inflammatory state, with increased receptor activator of nuclear factor kappa-B ligand signaling and elevated interleukin-1, interleukin-6, and tumor necrosis factor-α driving rapid bone turnover [13,14].

Bisphosphonates and denosumab remain effective, but some patients decline medication and look for structured alternatives. Mechanical loading is one of the major biological regulators of bone remodeling, and brief, high-intensity osteogenic loading has been proposed as a way to deliver sufficient skeletal strain in a controlled setting [15]. Clinical evidence in breast cancer survivorship remains sparse. We describe a breast cancer survivor with postmenopausal osteoporosis who showed measurable improvement in bone mineral density over approximately 16 months on a high-adherence lifestyle program incorporating supervised osteogenic loading, progressive resistance training, dietary modification, and supplementation, without anti-resorptive therapy.

## Case presentation

A 55-year-old postmenopausal woman presented for evaluation of bone health following routine screening. She had been diagnosed at age 36 years with high-grade ductal carcinoma of the right upper outer quadrant, clinical stage T2N2M0, with a 4-cm primary lesion and bulky nodal disease. Receptors were estrogen receptor-positive at 99%, progesterone receptor-positive at 20%, and human epidermal growth factor receptor 2-positive. Positron emission tomography/computed tomography showed no distant disease, and molecular testing was negative for breast cancer susceptibility gene 1 and gene 2 mutations.

Treatment consisted of neoadjuvant anthracycline/cyclophosphamide followed by a taxane and trastuzumab, then modified radical mastectomy. She completed one year of trastuzumab and comprehensive regional nodal irradiation. Endocrine therapy was tamoxifen for five years, followed by a three-month trial of ovarian suppression with leuprolide and letrozole that was discontinued for intolerance. Menses resumed with regular heavy cycles after ovarian suppression was stopped. Tamoxifen was then resumed for five further years, for a total of 10 years of therapy. Menopause occurred at age 52 years. She had no prior fragility fracture, no tobacco or alcohol use, no family history of osteoporotic fracture, and a body mass index of 22.8 at diagnosis.

**Figure 1:**
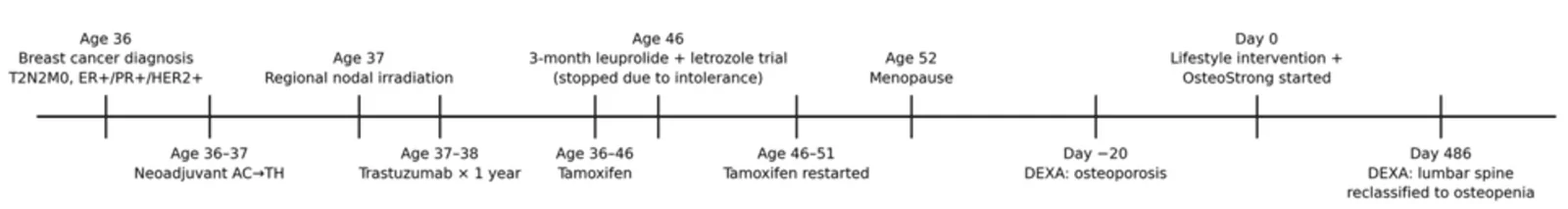
Clinical timeline from breast cancer diagnosis to bone mineral density follow-up This image was created using Python (matplotlib). ER, estrogen receptor; PR, progesterone receptor; HER2, human epidermal growth factor receptor 2; DEXA, dual-energy X-ray absorptiometry

To protect patient confidentiality, calendar dates in this report are expressed relative to Day 0, defined as the start of the structured lifestyle intervention; dates originally documented only to the month are anchored to the 15th of that month. Bone density was followed across three time points (age 41, Day -20, and Day 486; Figure 1). A scan at age 41 years, while she was premenopausal, demonstrated bone density within the expected range for age (lumbar spine 0.878 g/cm², *Z*-score -1.2; left total hip 0.781 g/cm², *Z*-score -1.1; left femoral neck 0.683 g/cm², *Z*-score -1.2). A repeat scan on Day -20 demonstrated osteoporosis: total lumbar spine bone mineral density 0.775 g/cm² (*T*-score -2.5), femoral neck 0.615 g/cm² (*T*-score -2.1), and total hip 0.713 g/cm² (*T*-score -1.9). Segmental analysis showed the greatest deficits at L3 and L4, each with a *T*-score of -3.0. The Fracture Risk Assessment Tool 10-year probability of major osteoporotic fracture was 7.8% and of hip fracture 0.9%. Alendronate was offered, but the patient declined because of concern about adverse effects [15].

Intervention

Beginning on Day 0, the patient initiated a structured lifestyle program: weekly supervised osteogenic loading at an OsteoStrong® center (OsteoStrong Franchising, Inc., Houston, TX), 10 to 15 minutes per session. The system uses four trigger devices applying brief, high-intensity, near-isometric axial loading - chest press (upper extremity), leg press (lower extremity), core pull (core), and a vertical lift that loads the spine in a manner similar to a deadlift. No dedicated interval imaging (e.g., bone scan or repeat positron emission tomography/computed tomography) was performed specifically to exclude skeletal metastases before initiation; the decision to proceed was based on her being 19 years from diagnosis with long-standing no evidence of disease and the absence of new bone pain, neurologic symptoms, or other findings suggestive of recurrence. No formal, predefined criteria for interrupting sessions and triggering further evaluation were established before the intervention began. Load progression at each station was governed by the device's own force feedback and by the patient's subjective tolerance from session to session, and she did not develop new or worsening bone pain, night pain, or other red-flag symptoms over the course of the intervention. Between Day 28 and Day 530 she completed 73 sessions, averaging one per week. Baseline force values were 374 lbs (2.9 × body weight) at chest press, 879 lbs (6.8 × body weight) at leg press, 343 lbs (2.6 × body weight) at core pull, and 446 lbs (3.4 × body weight) at vertical lift; values are reported as multiples of body weight, the metric the platform uses to normalize loading. Additional training included resistance training four times weekly with progressive overload (~240 minutes/week), moderate-to-vigorous aerobic exercise five times weekly (~300 minutes/week), and yoga, flexibility, and daily balance work (~2 hours/week). Diet shifted to a whole-food, plant-forward pattern emphasizing fruits, vegetables, legumes, whole grains, nuts, and olive oil, with calcium-rich foods prioritized daily and minimal red meat or ultra-processed foods. Supplementation included calcium 1000 mg, vitamin D 2000 international units, magnesium 200 mg, and omega-3 fatty acids 930 mg daily. Protein intake was intentionally increased to a target of 120 g per day.

Outcomes

**Figure 2:**
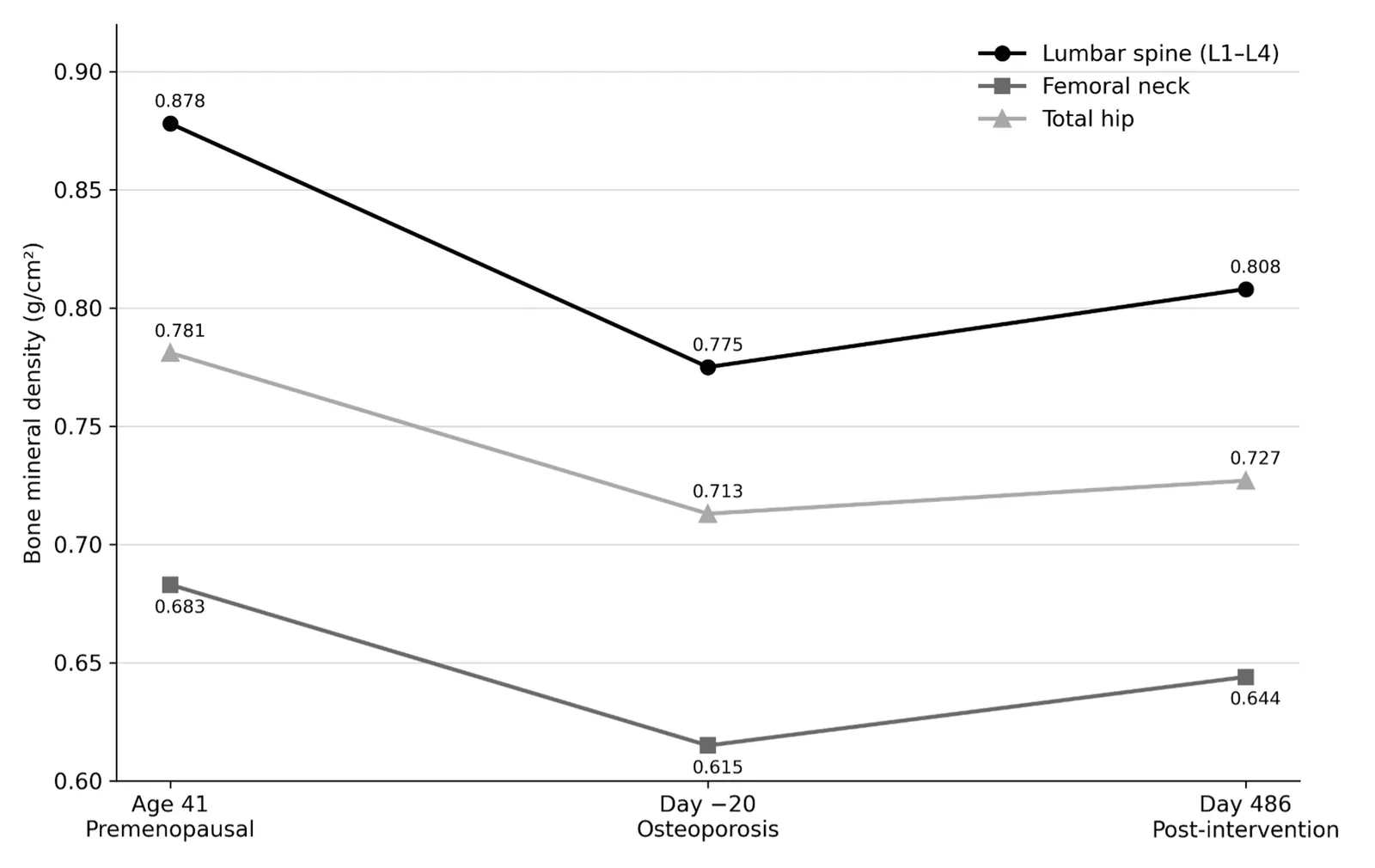
Serial bone mineral density measurements at major skeletal sites.

**Figure 3:**
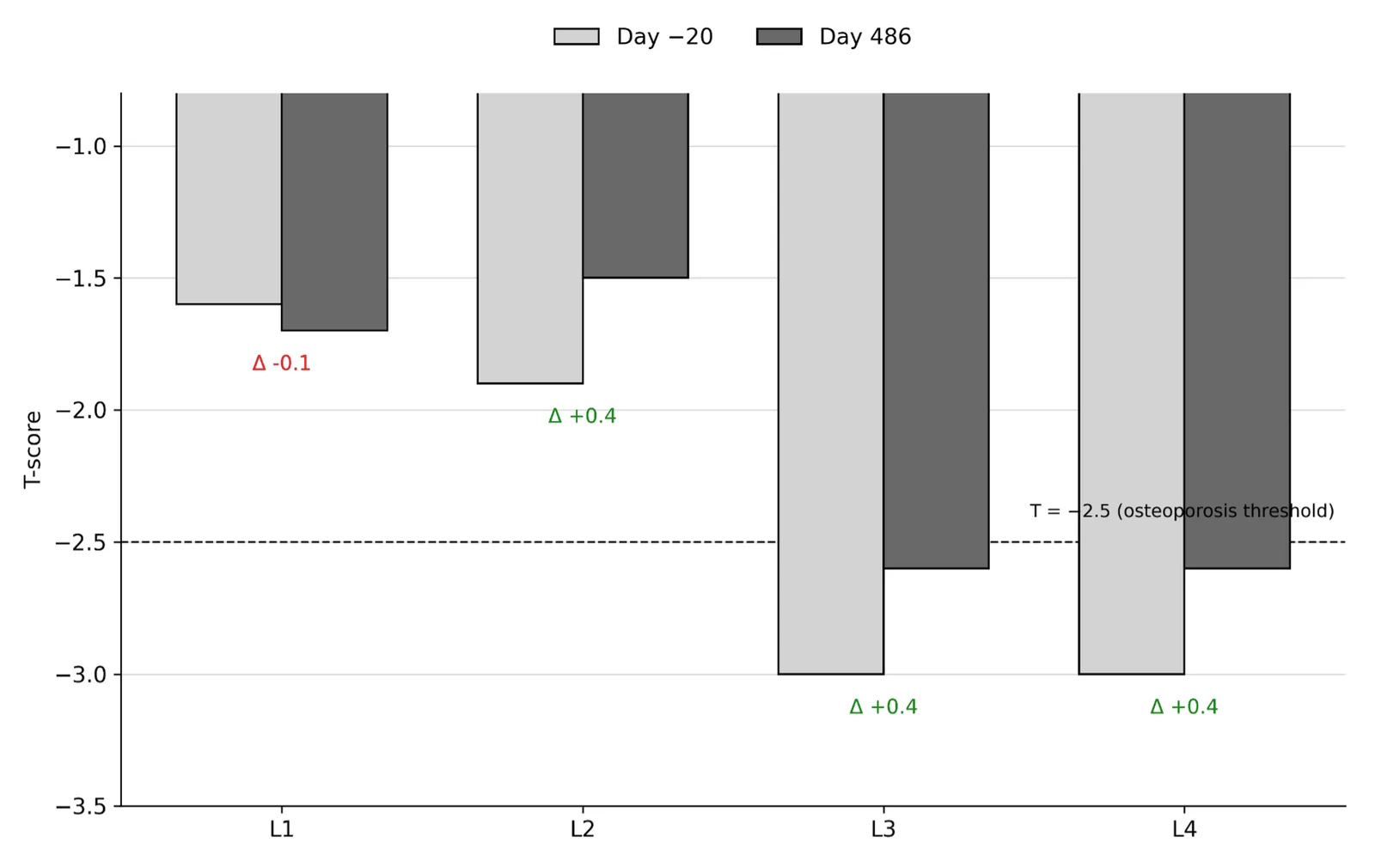
Lumbar vertebral T-scores before and after intervention.

**Figure 4:**
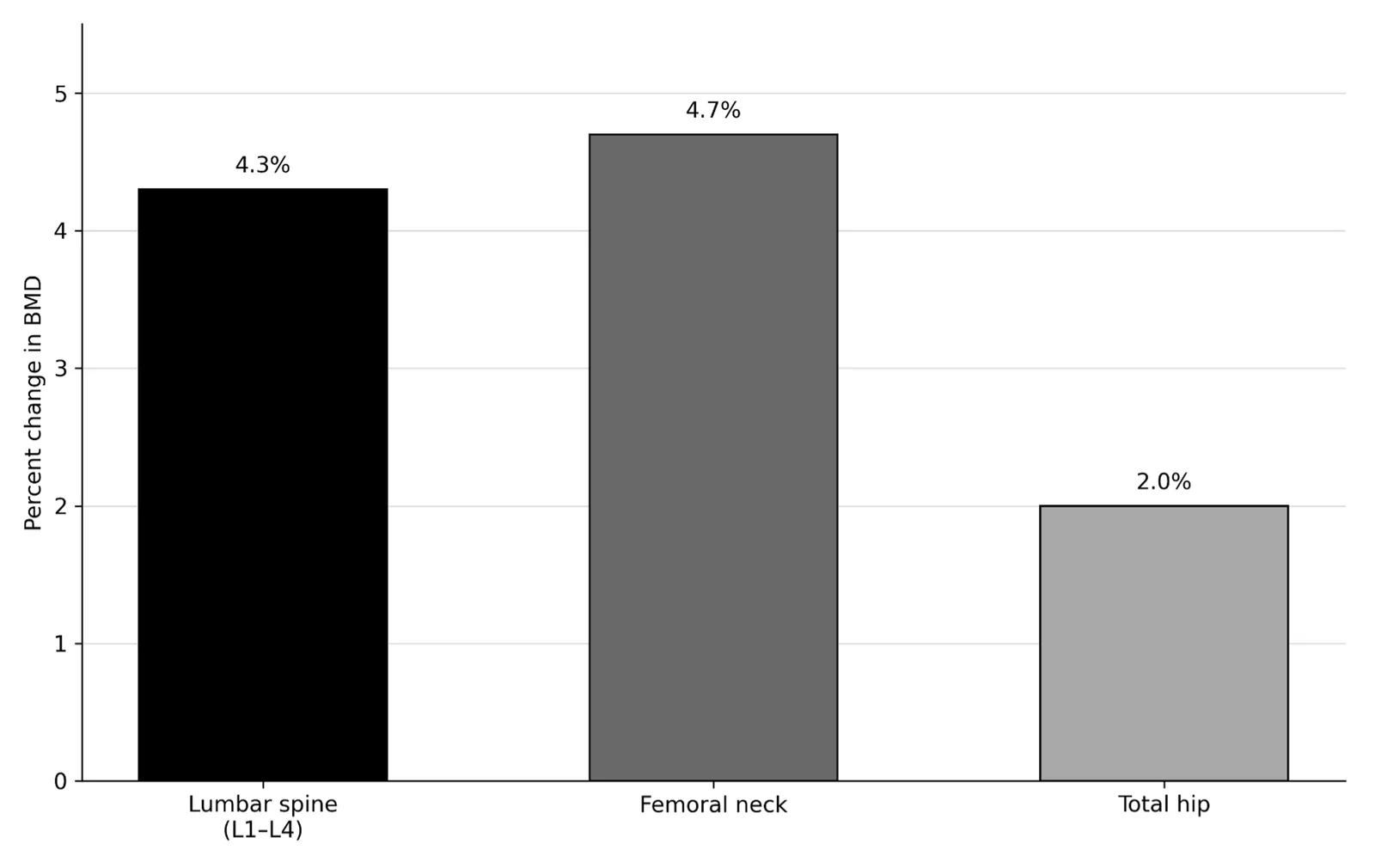
Percent change in bone mineral density from Day -20 to Day 486.

A follow-up scan on Day 486 (Tables 1-2; Figures 2, 3, 4) demonstrated improvement across measured sites. Total lumbar spine bone mineral density rose from 0.775 to 0.808 g/cm² (+4.3%; *T*-score -2.2), femoral neck from 0.615 to 0.644 g/cm² (+4.7%; *T*-score -1.8), and total hip from 0.713 to 0.727 g/cm² (+2.0%; *T*-score -1.8). The interpreting radiology report described osteopenia on the Day 486 study and confirmed a 4.2% lumbar and 4.7% femoral neck increase since Day -20, with no significant change at the total hip. Fracture Risk Assessment Tool probability fell to 7.0% (major fracture) and 0.8% (hip fracture). The most severely affected vertebrae recovered the most: L3 and L4 each moved from a *T*-score of -3.0 to -2.6. Of the 73 total osteogenic loading sessions completed by Day 530, 61 had been completed by the Day 486 follow-up scan, representing the exposure that preceded the observed bone mineral density changes. Osteogenic loading data are summarized in Table 3: overall improvement was 86% across the four stations, with chest press +97% (2.9 to a sustained 5.7 × body weight), leg press +125% (peak 15.2 × body weight), vertical lift +104% (peak 7.0 × body weight), and core pull +17% (peak 3.1 × body weight). Serum calcium and 25-hydroxyvitamin D remained within the reference range throughout follow-up (Table 4). 

**Table 1:** Serial dual-energy X-ray absorptiometry values and changes (postmenopausal, Day -20 to Day 486). *T*-scores apply to postmenopausal scans. *T*-score = standard deviations from the young-adult reference mean. BMD, bone mineral density

Region	Day -20 BMD (g/cm²)	Day 486 BMD (g/cm²)	% change	Day -20 *T*-score	Day 486 *T*-score	Δ*T*
L1	0.809	0.804	-0.6%	-1.6	-1.7	-0.1
L2	0.814	0.860	+5.7%	-1.9	-1.5	+0.4
L3	0.753	0.801	+6.4%	-3.0	-2.6	+0.4
L4	0.732	0.773	+5.6%	-3.0	-2.6	+0.4
Total L1-L4	0.775	0.808	+4.3%	-2.5	-2.2	+0.3
Femoral neck	0.615	0.644	+4.7%	-2.1	-1.8	+0.3
Total hip	0.713	0.727	+2.0%	-1.9	-1.8	+0.1

**Table 2:** Premenopausal baseline bone mineral density (age 41). *Z*-score = standard deviations from the age-matched reference mean, reported because the patient was premenopausal at baseline. BMD, bone mineral density

Region	BMD (g/cm²)	*Z*-score
Lumbar spine (L1-L4)	0.878	-1.2
Left total hip	0.781	-1.1
Left femoral neck	0.683	-1.2

**Table 3:** OsteoStrong® (OsteoStrong Franchising, Inc., Houston, TX) osteogenic loading performance progression (73 sessions, Day 28-Day 530). Data sourced from the OsteoStrong® platform performance records. ×BW, multiples of body weight

Station	First (lbs)	First (×BW)	Best (lbs)	Best (×BW)	Last (lbs/ ×BW)	% Improvement
Upper trigger (Chest Press)	374	2.9	736	5.7	736 / 5.7	+97%
Lower trigger (Leg Press)	879	6.8	1,979	15.2	1,468 / 11.3	+125%
Core trigger (Core Pull)	343	2.6	401	3.1	358 / 2.8	+17%
Postural trigger (Vertical Lift)	446	3.4	908	7.0	849 / 6.5	+104%
Overall average	-	-	-	-	-	+86%

**Table 4:** Routine laboratory values at baseline and follow-up. 25-hydroxyvitamin D = the major circulating metabolite used clinically to assess vitamin D status. Day 379 corresponds to the October follow-up laboratory draw, approximately 3.5 months before the Day 486 follow-up dual-energy X-ray absorptiometry scan. Reference ranges reflect the reporting laboratory’s standard intervals. Both analytes remained within range at both time points.

Test	Day -20 result	Day 379 result	Units	Reference range
Serum calcium	10.0	9.8	mg/dL	8.5-10.5
Serum 25-hydroxyvitamin D	51.5	52.4	ng/mL	25-80

## Discussion

The burden of osteoporosis and fragility fracture continues to rise, with annual care costs estimated in the tens of billions of dollars [16]. In breast cancer survivors, particularly those exposed to chemotherapy, prolonged endocrine therapy, and premature menopause, the clinical task is harder still. In our patient with hormone receptor-positive disease, systemic hormone replacement therapy was not an option despite Women’s Health Initiative data showing that hormone therapy reduces vertebral and non-vertebral fractures in women aged 50 years and older [4]. Although menses returned after chemotherapy and a brief course of ovarian suppression, the residual skeletal effects of prior amenorrhea, cytotoxic chemotherapy, and the antagonistic premenopausal effect of tamoxifen [11] do not appear to have been fully reversed, and she progressed from age-expected bone density to overt postmenopausal osteoporosis.

The central finding is that bone mineral density improved substantially over roughly 16 months with lifestyle modification alone. Lumbar spine rose by 4.3%, femoral neck by 4.7%, and total hip by 2.0%. Published dual-energy X-ray absorptiometry precision data suggest least significant change thresholds of approximately 2% to 3% at the spine and 3% to 5% at the hip [17-19]. Facility-specific precision and least significant change values were not available for the scanner used in this case, so these literature-derived thresholds cannot establish with certainty that the observed changes exceeded measurement variability. The lumbar spine change (+4.3%) exceeds even the upper end of the published spine threshold, making measurement noise a less likely explanation there; the femoral neck change (+4.7%) should be interpreted more cautiously given the comparatively poorer reproducibility typically reported at that site, and could in part reflect precision error rather than a true biological change. The 0.3-point gain in lumbar spine *T*-score falls within the range of early *T*-score improvement reported with first-line pharmacologic therapy over one to two years [19]. The pattern of recovery strengthens that interpretation: the most severely affected vertebrae recovered the most, with L3 and L4 each moving from -3.0 to -2.6 (Figure 3). Large gains at the most-affected sites and smaller gains elsewhere are difficult to reconcile with simple measurement variability

The Fracture Risk Assessment Tool fell only modestly, from 7.8 to 7.0%, which is expected: it estimates 10-year probability, often underestimates short-term change after bone mineral density improvement [20], has known limitations in women under 65 years [4], and is not recommended for treatment monitoring [16]. Trabecular bone score, which adds information about bone microarchitecture and refines fracture risk beyond *T*-score [16], was not reported here - a limitation. Even so, each 1% rise in spine bone mineral density has been associated with roughly a 1.5 to 2% relative reduction in vertebral fracture risk [21], so the densitometric gains are likely clinically meaningful.

Mechanically, the result is consistent with Wolff’s law: bone adapts to applied load [22]. The OsteoStrong protocol applies brief, near-isometric, axial compressive force at four anatomic sites in a single weekly 15-minute session. A prospective case series in peri- and postmenopausal women using a similar protocol reported modest gains in lumbar spine bone mineral density and trabecular bone score and proposed a minimum effective strain of approximately 4.2 × body weight at the upper skeleton, although several findings did not survive Bonferroni correction [23,24]. The available evidence supports biological plausibility but does not yet establish osteogenic loading as a proven therapy. In our case, the longitudinal force data add mechanistic support: upper extremity load advanced from 2.9 to 5.7 × body weight, crossing the proposed threshold well before the follow-up scan and holding above it for the remainder of the study, while lower extremity and postural loads reached 15.2 and 7.0 × body weight, well above any appendicular threshold.

The non-OsteoStrong exercise was not incidental. Moderate-to-high-load resistance training improves bone mineral density in postmenopausal women, with one meta-analysis reporting an average gain of 1.82% in active participants compared with little change in controls [25]. A separate meta-analysis of 15 randomized trials found that exercise reduces fall-related fractures by about 40% [26]. Roughly 90% of hip fractures result from falls [27], so balance and mobility work are better understood as fracture prevention than as wellness add-ons. Current guidance recommends musculoskeletal strengthening and balance training but is often vague about dose, duration, and intensity [28], and this case supports a more structured application of those principles. The dietary changes followed American College of Lifestyle Medicine recommendations [29], with daily calcium-rich foods, calcium and vitamin D supplementation, and a 120 g daily protein target. The gut-bone axis is another plausible contributor: gut microbiota influence calcium and magnesium absorption, short-chain fatty acid signaling at the osteoblast level, and systemic inflammation [30,31]. A single case cannot establish a microbiome-mediated effect, but these mechanisms help explain how a combined dietary and exercise intervention might shift skeletal remodeling.

Limitations include the single-patient design, the deliberately multi-component intervention that precludes isolation of any single element, the absence of facility-specific dual-energy X-ray absorptiometry precision data, the lack of trabecular bone score reporting, and the absence of dedicated pre-intervention imaging to exclude skeletal metastases or a predefined, symptom-triggered stopping protocol for the osteogenic loading program. Occult skeletal metastatic disease, although statistically unlikely at this disease-free interval, cannot be fully excluded without imaging, and axial loading of an undetected lesion could theoretically increase fracture risk; future application of this protocol in cancer survivors would benefit from baseline imaging where clinically indicated and an explicit symptom-triggered stopping rule, with risk stratification based on factors such as disease-free interval, prior sites of disease, and any interval symptoms, guiding how much of this workup is warranted before initiating high-intensity axial loading in a given patient. Even so, the magnitude of change, the regional pattern of recovery, and the reclassification of the lumbar spine from osteoporosis to osteopenia make the result clinically compelling and worth further study.

## Conclusions

This case describes a breast cancer survivor with treatment-associated postmenopausal osteoporosis whose bone mineral density improved meaningfully over roughly 16 months on a structured, high-adherence lifestyle program - supervised osteogenic loading, progressive resistance and aerobic training, balance and mobility work, dietary optimization, and supplementation - without anti-resorptive therapy. The lumbar spine moved from the osteoporotic to the osteopenic range, with concordant improvement at the femoral neck. No single element of the program can be credited in isolation, but the improvement is large enough to justify a closer look at structured, multidisciplinary, non-pharmacologic strategies in survivorship care, especially for patients with hormone receptor-positive disease who have few hormonal treatment options left.
